# Blood Pressure Increase following COVID-19 Vaccination: A Systematic Overview and Meta-Analysis

**DOI:** 10.3390/jcdd9050150

**Published:** 2022-05-09

**Authors:** Fabio Angeli, Gianpaolo Reboldi, Monica Trapasso, Gabriella Santilli, Martina Zappa, Paolo Verdecchia

**Affiliations:** 1 Department of Medicine and Surgery, University of Insubria, 21100 Varese, Italy; marty-italy92@hotmail.it; 2 Department of Medicine and Cardiopulmonary Rehabilitation, Istituti Clinici Scientifici Maugeri IRCCS, 21049 Tradate, Italy; 3 Department of Medicine, and Centro di Ricerca Clinica e Traslazionale (CERICLET), University of Perugia, 06100 Perugia, Italy; paolo.reboldi@unipg.it (G.R.); santilli.gabriella@gmail.com (G.S.); 4 Dipartimento di Igiene e Prevenzione Sanitaria, PSAL, Sede Territoriale di Varese, ATS Insubria, 21100 Varese, Italy; montrapasso@gmail.com; 5 Fondazione Umbra Cuore e Ipertensione-ONLUS and Division of Cardiology, Hospital S. Maria della Misericordia, 06100 Perugia, Italy; verdecchiapaolo@gmail.com

**Keywords:** COVID-19, vaccine, blood pressure, hypertension, adverse drug reaction, BNT162b2, mRNA-1273, Ad26.COV2.S, CVnCoV, ChAdOx1nCoV-19, NVX-CoV2373, Gam-COVID-Vac

## Abstract

Coronavirus disease 2019 (COVID-19) vaccines proved a strong clinical efficacy against symptomatic or moderate/severe COVID-19 and are considered the most promising approach for curbing the pandemic. However, some questions regarding the safety of COVID-19 vaccines have been recently raised. Among adverse events to vaccines and despite a lack of signal during phase III clinical trials, an increase in blood pressure (BP) after COVID-19 vaccination has been reported as a potential adverse reaction. We systematically analyze this topic and undertook a meta-analysis of available data to estimate the proportion of patients with abnormal BP or raise in BP after vaccination. Six studies entered the final analysis. Overall, studies accrued 357,387 subjects with 13,444 events of abnormal or increased BP. After exclusion of outlier studies, the pooled estimated proportion of abnormal/increased BP after vaccination was 3.20% (95% CI: 1.62–6.21). Proportions of cases of stage III hypertension or hypertensive urgencies and emergencies was 0.6% (95% CI: 0.1% to 5.1%). In conclusion, abnormal BP is not rare after COVID-19 vaccination, but the basic mechanisms of this phenomenon are still unclear and require further research.

## 1. Introduction

Different therapeutic strategies are under scrutiny to block the transition from infection to severe forms of coronavirus disease 2019 (COVID-19) [1,2]. They include prevention of the viral RNA synthesis and replication, blockade of SARS-CoV-2 from binding to human cell receptors, the restoration of the host’s innate immunity, and the modulation of the host’s specific receptors or enzymes [1,2,3,4,5,6].

However, vaccines to prevent SARS-CoV-2 infection are considered the most promising approach, offering the opportunity to come out of the current phase of the pandemic [2,7]. 

COVID-19 vaccines have been developed using different advanced technologies and several platforms [7,8,9], including live attenuated vaccines, inactivated vaccines, recombinant protein vaccines, vector vaccines (replication-incompetent vector vaccines, replication-competent vector vaccines, and inactivated virus vector vaccines), DNA vaccines, and RNA vaccines (Table 1). By 18 March 2022 a total of 10,925,055,390 vaccine doses have been administered globally (5,007,662,851 persons vaccinated with at least one dose, and 4,446,884,806 persons fully vaccinated; (https://covid19.who.int/ accessed on 18 March 2022).

Despite the clinical efficacy against symptomatic or moderate/severe COVID-19 ranged from 67% to 95% in several clinical trial [10,11,12,13,14,15], some questions regarding the safety of COVID-19 vaccines have been recently raised and mainly based on reports of thromboembolic events [16,17,18,19,20,21,22]. An extremely carefully monitoring of safety issues showed other rare adverse events occurring after COVID-19 vaccination, including anaphylaxis, myocarditis/pericarditis, and Guillain-Barré Syndrome (https://www.cdc.gov/coronavirus/2019-ncov/vaccines/safety/adverse-events.html accessed on 18 March 2022) [2].

Just recently and despite a lack of signal during the main phase III clinical trials, an increase in blood pressure (BP) after COVID-19 vaccination has been reported [2,23,24,25].

The main aim of this review was to systematically analyze data on this topic, offering an overview of the clinical implications and potential mechanisms of this phenomenon. Specifically, we undertook a meta-analysis of available data to estimate the proportion of patients with abnormal or raised BP after vaccination. 

## 2. Materials and Methods

### 2.1. Study Selection and Outcome Measures

We addressed analyses and clinical studies (both retrospective and prospective) meeting all the following inclusion criteria: (a) data on incidence of abnormal or increased BP regardless of the specific vaccination strategy; (b) publication in a peer-reviewed journal before 28 February 2022; (c) no age or language restriction, in order to avoid discriminating papers not written in English (“tower of Babel bias”) [26]. 

### 2.2. Data Sources and Searches

Candidate studies were searched through MEDLINE, Scopus, Web of Science, and CINHAL, using research Methodology Filters [27]. The following research terms were used: “SARS-CoV-2”, “COVID-19”, “2019-ncov”, “coronavirus”, “blood pressure”, “hypertension”, and “adverse events”. We made a further screening of review articles, published proceedings of conferences, and regulatory agencies files [28] in order to identify other relevant studies. 

### 2.3. Data Synthesis

**Figure 1:**
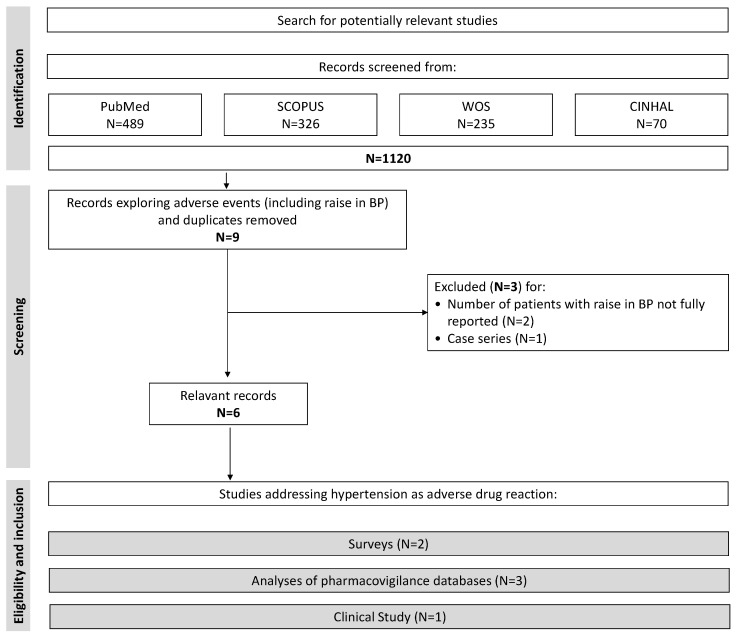
Criteria used for selection of studies.

Table 2 shows the clinical studies identified on the basis of the above criteria. Overall, studies accrued 357,387 subjects. Figure 1 shows the flow diagram with the criteria used for selection of studies. We used the Preferred Reporting Items for Systematic Reviews and Meta-Analyses (PRISMA) statement (Table S1) [29]. Data were independently extracted by two authors (FA and PV). Disagreements were discussed in conference.

Three reviewers independently assessed the risk of bias of each of the included studies and discussed their assessments to achieve consensus. The included studies were scored for quality using the Newcastle-Ottawa scale. The scale items assess appropriateness of research design, recruitment strategy, response rate, representativeness of sample, objectivity/reliability of outcome determination, power calculation, and appropriate statistical analyses [30,31]. Score disagreements were resolved by consensus and a final agreed-upon rating was assigned to each study (Table S2) [30,31].

### 2.4. Data Analysis

Proportions were calculated by dividing the number of patients with the specific endpoint by the total number of patients for each study. We used a generalized linear mixed model (GLMM)—i.e., a random intercept logistic regression model—for the meta-analysis of proportions [32]. We also tested for the presence of statistical outliers using the method described by Harrer et al. [33]. Studies are defined as outliers when their 95% confidence interval lies outside the 95% confidence interval of the pooled effect.

The null hypothesis of homogeneity across individual studies was tested by using the Q test. Pooled estimates were assessed for heterogeneity by using the I^2^ statistic [34]. 

Analyses were performed using R version 4.1.3 (R Foundation for Statistical Computing, Vienna, Austria) and Stata, version 16 (StataCorp LP, College Station, TX, USA). 

## 3. Results

Literature search initially yielded 1120 reports. After removal of duplicates and studies not focused on safety of COVID-19 vaccines, we reviewed nine clinical studies in full text [24,25,35,36,37,38,39,40,41]. 

We excluded two studies because of lack of data on the precise number of adverse drug reactions (ADRs) or a clear definition of raise in BP [37,39]. Among the remaining seven studies, we removed one study reporting a case series of vaccinated patients (Figure 1) [24]. Thus, six studies entered the final analysis (Table 2) [25,35,36,38,40,41]. Of these, two were cross-sectional surveys [25,40], three analyzed data from pharmacovigilance databases [36,38,41], and one evaluated BP after 15 min from vaccination among a cohort of patients and healthcare workers [35]. 

### 3.1. Excluded Studies

Meylan and co-workers reported a case series of nine patients with stage III hypertension documented within minutes of vaccination, of which eight were symptomatic [24] (Table S3). BP was measured with an oscillometric validated manometer with at least three sets of separate values at 5-min intervals [24]. Median age was 73 years, and eight of nine patients had a history of arterial hypertension with most patients on antihypertensive therapy [24]. All but one patient received the BNT162b2 vaccine. Of note, patients had a previous well controlled hypertension. All patients recovered but required at most several hours of monitoring at tertiary center’s emergency department [24].

Sanidas and co-workers [37,39] (Table S3) investigated the effects of vaccination on BP in patients with known hypertension and healthy controls. A total of 100 patients between the age of 50 to 70 years old were included [37,39]. They were randomly assigned to one of the approved and available vaccines (BNT162b2, mRNA-1273, Ad26.COV2.S, and ChAdOx1nCoV-19) [37,39]. All participants had systolic BP < 140 mmHg and diastolic BP < 90 mmHg before vaccination and volunteered for home BP measurements and ambulatory BP measurements between the 5th and the 20th day after fully COVID-19 vaccination [37,39]. Patients with known history of hypertension showed a mean home BP equal to 175/97 mmHg. Similar results were also recorded for 24-h mean BP (177/98 mmHg) [37,39]. Healthy controls showed a BP of 158/96 mmHg and 157/95 mmHg during home and ambulatory monitoring, respectively [37,39]. Five of 50 hypertensive patients received additional medication whereas some of the non-hypertensive patients started life modification changes and systematic BP measurements for a possible diagnosis of hypertension [37,39].

Finally, a recent analysis by Ch’ng and coworkers [37,39] (Table S3) collected data from 4906 healthcare workers. BP was measured three times for each staff member using an automated BP monitor. Pre-vaccination BP was recorded when the staff members arrived at the vaccination site; post-vaccination BP was measured immediately after vaccination and 15–30 min later in a waiting room [37,39]. Mean pre-vaccination systolic and diastolic BP were 130.1 mmHg and 80.2 mmHg, respectively. When compared with baseline, BP was increased in more than half of the subjects immediately and 30 min post vaccination. The mean changes immediately after vaccination were +2.3/2.4 mmHg for systolic/diastolic BP [37,39]. 

### 3.2. Included Studies

The retrospective analysis by Bouhanick and co-workers, describing the prevalence of high BP after vaccination, exhibited the largest proportion of this phenomenon [35]. They retrospectively investigated BP profile of vaccinated patients and healthcare workers to describe the course of BP values after the first and the second injection of vaccine and to assess the prevalence of high BP values in this population. Notably, BP was measured 15 min after vaccine injection and measurements were performed with a validated automatic electronic device [35]. A total of 21,909 subjects had complete data on BP (61.7% were women, mean age was 59 years). Among these subjects, 8121 people (37.1%) exhibited systolic and/or diastolic BP above 140 and/or 90 mmHg after the first injection. Among the subjects with high BP after the first injection, 64% were still hypertensive after the second one [35].

Interrogations of pharmacovigilance databases [36,38,41] showed proportions of abnormal or increased BP after vaccination ranging from 0.93% to 2.89%. 

Proportions from surveys, specifically designed to evaluate BP changes after vaccination, was about 5% (5.06% in the analysis by Tran and co-workers [40] and 5.31% in the sample from Angeli and co-workers [25]).

More specifically, the Italian prospective survey [25] showed that among 113 health care workers who received the Pfizer vaccine, 6 subjects (5.3%) showed a rise in systolic or diastolic BP at home ≥ 10 mmHg during the first five days after the first dose of the vaccine when compared with the five days before the vaccine (the BP rise required an intensification of BP-lowering treatment in 4 subjects) [25]. Interestingly, the subjects with documented infection by SARS-COV-2 over the previous year showed a higher frequency of systemic reactions to vaccine when compared with those without history of documented infection (38% vs. 10%, *p* = 0.004). History of COVID-19 was associated with a higher incidence of rise in BP when compared with subjects without previous exposure to SARS-CoV-2 (23% vs. 3%, *p* = 0.002). Symptomatic tachycardia was noted in 7 and 3 respondents after the first and second dose of vaccine, respectively, and there were no cardiovascular events or severe or immediate allergic reactions during a follow-up of 103 days [25].

Similarly, Tran and co-workers performed a cross-sectional survey including 1028 subjects (899 had one ChAdOx1nCoV-19 dose and the rest received 2 doses) [40]. Abnormal BP after vaccination was recorded in 52 subjects. 

Quality assessment of the included studies is reported in Table S2. 

### 3.3. Pooled Analyzses

**Figure 2:**
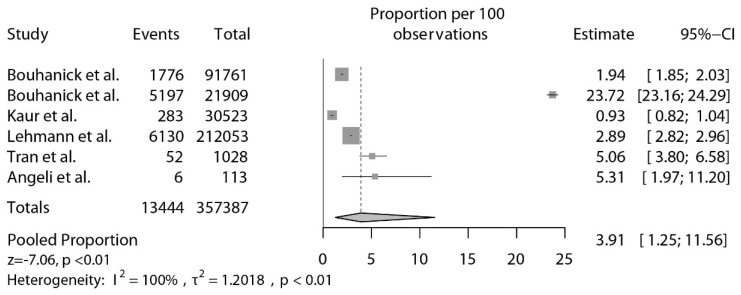
Proportions of increased BP after vaccination [25,35,36,38,40,41].

**Figure 3:**
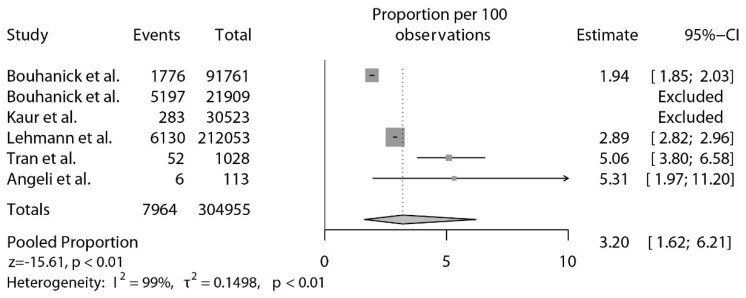
Proportions of increased BP after vaccination, after the exclusion of outlier studies [25,35,36,38,40,41].

Overall, the pooled estimated proportion of abnormal or increased BP after vaccination was 3.91% (95% confidence interval [CI]: 1.25–11.56, *p* < 0.01; Figure 2). Nonetheless, two studies were identified as statistical outliers [35,38]. As depicted in Figure 3, after the exclusion of these 2 studies [35,38], the pooled proportion of abnormal or increased BP after vaccination was 3.20% (95% CI: 1.62–6.21, *p* < 0.01).

We also evaluated the proportion of cases of stage III hypertension or hypertensive urgencies and emergencies. Four studies reported the proportion of patients who developed these outcomes after COVID-19 vaccination (range: 0.1% to 3.2%). The pooled proportion of these events was 0.6% (95% CI: 0.1–5.1%). 

## 4. Discussion

To the best of our knowledge, this is the first systematic review designed to investigate the occurrence of abnormal or increased BP after COVID-19 vaccination. The main novelty of our study is the evidence that a raise in BP after COVID-19 vaccination is not unusual. The proportions of patients with abnormal BP or with a significant increase in BP ranged from 0.93% to 23.72%, with a pooled point estimate of 3.91% (3.20% excluding statistical outliers). Moreover, the estimate of stage III hypertension or hypertensive urgencies and emergencies following COVID-19 vaccination was 0.6% (95% CI: 0.1–5.1%).

As aforementioned, the design of the study largely affected such proportions, with the highest value recorded in a retrospective study carried out in healthcare workers who received the BNT162b2 vaccine in a University Hospital in Toulouse [35]. Specifically, Bouhanick and co-workers [35] reported the course of BP after the injection of vaccine and assessed the incidence of high BP values in this population [35]. BP was measured 15 min after vaccination in all patients who received a first or a second injection. Subjects remained seated for 15 min after injection, and hypertension was defined as BP greater than or equal to 140/90 mmHg (grade III hypertension was declared if BP was greater than or equal to 180/111 mmHg) [35]. As remarked by the authors, the main limitation of this study was the lack of pre-vaccination control of BP and, thus, the proportion of subjects with high BP observed after the injection may reflect an unknown or insufficiently controlled hypertension [35]. 

Conversely, analyses of pharmacovigilance databases and clinical surveys, showed rates of abnormal BP or significantly increased BP after vaccination ranging from 0.93% to 2.89% (Figure 2). 

The precise basic mechanism of this phenomenon is still unclear and further studies are required to investigate the association between COVID-19 vaccination and hypertension [2,23,42]. Stress response, white-coat effect, and the possible role of excipients [24] might contribute to explain the high prevalence of abnormal BP values recorded immediately after vaccination. Nonetheless, the resulting features of COVID-19 vaccination resemble those of active COVID-19 disease [2,23,43,44]. It is well known that the entry of severe acute respiratory syndrome coronavirus 2 (SARS-CoV-2) occurs through the angiotensin-converting enzyme 2 (ACE2) receptors of the host cells [1,3,6,45,46,47,48]. Recent observations support the notion that when a vaccinated cell dies or are destroyed by the immune system, the debris may release a large amount of Spike proteins and protein fragments (free-floating Spike proteins) [2,23]. Spike proteins produced upon vaccination have the native-like mimicry of SARS-CoV-2 Spike protein’s receptor binding functionality and prefusion structure [49]. The native-like conformation of the Spike protein produced by vaccines has the potential to interact with ACE2, leading to its internalization and degradation [50]. The loss of ACE2 receptor activity from the outer layer of the cell membrane, as mediated by the interaction between ACE2 and SARS-CoV-2 Spike proteins, leads to less angiotensin II inactivation resulting from a reduced generation of antiotensin_1–7_. It is well known that angiotensin_1–7_ binds to the Mas receptor and reduces several effects of angiotensin 2 including inflammation, reabsorption of renal sodium, release of vasopressin and aldosterone, and fibrosis [46,47,51]. Thus, the imbalance between angiotensin II overactivity and of antiotensin_1–7_ deficiency after vaccination may trigger a raise in BP [45,46,47].

Our systematic review and meta-analysis has several limitations. First, studies included in our analysis did not use a control group to unmask the real effect of COVID-19 vaccination on BP and showed a low accounting comparability (Table S2). Second, and as aforementioned, time of BP recording (from 15 min to several days after vaccination) clearly affects the rates of BP increase after vaccination. Finally, pharmacovigilance databases provided the largest cohorts of subjects exploring this phenomenon. However, they analyzed the rates of BP increase as a self-reported phenomenon.

## 5. Conclusions

Vaccines to prevent SARS-CoV-2 infection elicit an immune neutralizing response, and they are the most promising approach for curbing the pandemic. 

However, some concerns regarding the safety of COVID-19 vaccines have been recently raised, including an increase in BP. Our systematic review and meta-analysis of observational studies specifically investigated this phenomenon.

Overall, included studies accrued 357,387 subjects with 13,444 events of abnormal or increased BP. The pooled proportions of abnormal/increased BP or stage III hypertension recorded following vaccination (3.20% and 0.6%, respectively) showed that this event should not be considered sporadic. However, in view of the small number of included studies and their inherent quality limitations (different times of observation, definition of BP increase, and a lack of a control group), the observed phenomenon requires further investigation in controlled settings. 

## Figures and Tables

**Table 1:** Main features of COVID-19 vaccines.

Vaccine	Developer	Platform	Doses
BNT162b2 *	Pfizer/BioNTech	mRNA	2
mRNA-1273 *	Moderna	mRNA	2
Ad26.COV2.S *	Janssen/Johnson & Johnson	DNA Adenovirus vector	1
CVnCoV	CureVAC	mRNA	2
ChAdOx1nCoV-19 *	AstraZeneca/University of Oxford/Serum Institute of India	DNA Adenovirus vector	2
NVX-CoV2373 *	Novavax	Recombinant protein	2
Gam-COVID-Vac (Sputnik V)	Gamaleya Institute	DNA Adenovirus vectors	2

* vaccines authorized for use in the European Union (https://www.ema.europa.eu/en/human-regulatory/overview/public-health-threats/coronavirus-disease-covid-19/treatments-vaccines/covid-19-vaccines accessed on 18 March 2022).

**Table 2:** Main features of studies included in the analysis.

Study	Source	Cohort (N)	Year	Vaccine	Outcome		Severe Increase in BP * (N)
					Definition	N	
Bouhanick et al. [36]	Pharmacovigilance database	91,761	2021	BNT162b2, ChAdOx1nCoV-19, Ad26.COV2.S	Abnormal BP	1776	-
Bouhanick et al. [35]	Patients and healthcare workers	21,909	2022	BNT162b2	Persistent BP ≥ 140/90 (15 min after vaccination)	5197	709
Kaur et al. [38]	Pharmacovigilance database	30,523	2021	BNT162b2, ChAdOx1nCoV-19, mRNA-1273	Abnormal BP	283	36
Lehmann et al. [41]	Pharmacovigilance database	212,053	2021	BNT162b2, ChAdOx1nCoV-19, Ad26.COV2.S, mRNA-1273	Abnormal BP	6130	551
Tran et al. [40]	Cross-sectional online survey	1028	2021	ChAdOx1nCoV-19	Self reported hypertension	52	-
Angeli et al. [25]	Cross-sectional online survey	113	2021	BNT162b2	Raise in home BP > 10 mmHg	6	2

* severe increase in BP included stage III hypertension, hypertensive urgencies, and hypertensive emergencies.

## Data Availability

The data underlying this article is fully reported in tables and figures.

## Supplementary Materials

The following supporting information can be downloaded at: https://www.mdpi.com/article/10.3390/jcdd9050150/s1, Table S1: PRISMA checklist; Table S2: Assessment of the quality of included studies using the Newcastle-Ottawa Scale; Table S3: Main characteristics of excluded studies.
